# Mapping the barriers and facilitators of oral healthcare access for vulnerable migrants across high-income countries: a scoping review

**DOI:** 10.1038/s41405-026-00398-0

**Published:** 2026-02-13

**Authors:** Zainab Lal, Luisa Silva, Nadia Alam, Mayuri Gogoi, Rebecca F. Baggaley, Pip Divall, Holly Reilly, Harriet Walter, Manish Pareek

**Affiliations:** 1 Division of Public Health and Epidemiology, University of Leicester, Leicester, UK; 2 Development Centre for Population Health, University of Leicester, Leicester, UK; 3 Warwick Medical School, University of Warwick, Coventry, UK; 4 Institute of Health Informatics, University College London, London, UK; 5 Education Centre Library, University Hospitals of Leicester NHS Trust, Leicester, UK; 6 Department of Genetics and Genome Biology, University of Leicester, Leicester, UK; 7 National Institute of Health Research (NIHR) Leicester Biomedical Research Centre (BRC), Leicester, UK; 8 Department of Infection and HIV Medicine, University Hospitals of Leicester NHS Trust, Leicester, UK; 9 National Institute of Health Research (NIHR) Applied Health Collaboration (ARC) East Midlands, Leicester, UK

**Keywords:** Dental public health, Dental epidemiology

## Abstract

**Background:**

According to the World Health Organisation, oral health (OH) diseases are a major global health issue and outcomes are consistently poorer among refugees and migrants than host populations in many high-income countries (HICs). In the UK, the Office for Health Improvement and Disparities recognises asylum seekers, refugees, undocumented migrants, low-wage migrants, unaccompanied minors, and victims of trafficking as vulnerable migrants. These groups face worse OH outcomes due to systemic, socio-economic, cultural, and lifestyle-related factors, alongside barriers to accessing dental services. This scoping review explores the barriers and facilitators to oral healthcare experienced by vulnerable migrants in HICs.

**Methods:**

We conducted a scoping review using the Arksey and O’Malley framework and reported findings in line with PRISMA-ScR. Embase and MEDLINE were searched from inception until April 30th 2024, for studies examining factors influencing access to oral healthcare services. Data were charted and thematically mapped onto the Dahlgren and Whitehead model of Social Determinants of Health (SDH).

**Results:**

Of 3894 identified records, 17 studies (10 qualitative, 5 quantitative, and 2 mixed-methods) were included, covering 2653 participants across 8 HICs (USA, UK, Australia, Austria, Germany, Finland, Saudi Arabia and Canada). Barriers and facilitators were present across all SDH layers. At the socio-economic, cultural, and environmental level, financial barriers were most commonly reported (12/17 studies). Language difficulties, low awareness of services, and mistrust of healthcare providers mapped to living and working conditions, while acculturation and social support aligned with the social and community networks layer. Limited knowledge of prevention was noted under lifestyle factors, and lastly, gender roles under personal characteristics. Cultural and religious norms also shaped care-seeking, with spirituality and religious traditions supporting positive OH practices.

**Conclusions:**

We identified barriers and facilitators to oral healthcare access across personal, behavioural, social, and structural levels, contextualised within the SDH framework. Addressing these requires policies and practices that address structural barriers, integrate OH into national public health strategies, and emphasise inclusive, culturally competent care to improve access to OH services for these groups.

## Background

Vulnerable migrant populations, including asylum seekers, refugees, undocumented migrants, low-wage migrant workers, unaccompanied minors, and victims of trafficking, experience a disproportionately higher burden of oral health disease compared to the host population in high-income countries (HICs) [1–3]. This is despite oral diseases, such as dental caries, periodontal disease, tooth loss, and oral cancer being largely preventable in nature [2, 4]. These disparities are well documented and are often compounded by complex health needs, poorer general and oral health outcomes, and limited access to healthcare [3, 5, 6]. These migrants encountering several vulnerabilities also experience a higher risk for oral disease influenced by forced displacement, exposure to conflict and trauma, precarious living conditions, and systemic barriers to accessing healthcare [3, 7–9].

A national oral health survey in Germany, reported significantly higher caries experience among refugees (mean = 3.13) compared to the host population (mean = 0.5) [10]. Another cross-sectional study found that 87.5% of surveyed refugees in Germany (*n* = 386) had untreated dental caries [11]. Poor oral health not only impairs basic functions such as eating and speaking but is also associated with systemic inflammatory conditions and chronic diseases such as cardiovascular disease, diabetes mellitus, Alzheimer’s disease, and respiratory infections [12–14]. The psychosocial consequences, including stigma, low self-esteem, and reduced quality of life, further highlight the impact of poor oral health. These consequences are exacerbated in migrant populations already facing multiple challenges.

Evidence suggests that refugees and asylum seekers underutilise oral healthcare services compared to the general population in HICs, reflecting structural barriers such as cost of services, lack of insurance coverage, language difficulties, and challenges navigating healthcare systems [3, 5, 6, 15, 16]. Barriers to accessing services contribute to untreated oral diseases significantly impacting their general and oral health-related quality of life [5, 6]. Socio-economic, cultural, and environmental factors also shape migrants’ perceptions, knowledge and behaviours around oral health, some of which may contribute to disparities in service access [17–19].

Previous studies have provided important insights into factors affecting oral health care among asylum seekers and refugees (ASRs), largely by using qualitative data [9, 16], and by broadly assessing oral health outcomes in these populations [3]. However, there remains a gap in synthesised quantitative and qualitative evidence that comprehensively maps the range of barriers and facilitators to oral healthcare access across diverse contexts. Our study addresses this by covering a broader population of vulnerable migrants including ASRs, undocumented migrants, socially disadvantaged migrant women, and low-paid migrant workers. Crucially, we utilise the Social Determinants of Health (SDH) model as a conceptual framework, allowing us to organise and analyse these findings at multiple systemic levels and to inform policy and practice in a structured manner [20]. In this review, migrants, according to the International Organisation of Migration are individuals who move away from their usual place of residence, either within or across international borders, temporarily or permanently, for various reasons [21].

## Methods

The Arksey and O’Malley methodological framework was utilised in this scoping review [22]. The study follows the Preferred Reporting Items for Systematic Review and Meta-analysis Extension for Scoping Reviews (PRISMA-ScR) [23].

### Search strategy and selection criteria

The search strategy was developed in consultation with PD, an academic librarian. Relevant keywords and Medical Subject Headings (MeSH) were identified and grouped under three main concepts including migrant populations, oral health conditions, and dental care professionals. Boolean operators (“AND”, “OR”) and truncations were used to combine and expand search terms. Searches were conducted in MEDLINE and Embase databases up to April 30, 2024. Details of the search strategy are provided in Supplementary Material 1. We included primary studies published in all languages of any design (qualitative, quantitative, or mixed-methods), conducted in HICs, as defined by the World Bank classification (gross national income (GNI) per capita exceeding USD $13,935) [24].

The target ‘population’ were vulnerable migrants, defined by the Office for Health Improvement and Disparities (OHID), Department of Health and Social Care (UK), to include groups such as asylum seekers, refugees, people who have been trafficked, undocumented migrants, and low-paid migrant workers as populations who may experience heightened vulnerabilities before, during, or after the migration journey [1]. We acknowledge, however, that the term “vulnerable migrants” can be reductive, and people who migrate are not defined by, nor should they be reduced to, their immigration status or associated experiences of trauma.

The ‘comparator’ was the host population in HICs; however, studies investigating the outcome among migrant groups alone were still included in the review. The main ‘outcome’ was barriers and facilitators to accessing oral healthcare. Studies were excluded if they reported on outcomes unrelated to access to oral healthcare, were case reports, editorials and reviews and conducted in settings other than HICs.

### Study screening

All studies were imported into Rayyan (web-based systematic literature search support tool, rayyan.ai) for de-duplication followed by subsequent screening. Four reviewers (ZL, LS, MG and RB) performed title and abstract screening. ZL screened all full texts of identified eligible studies. LS and NA reviewed full texts of studies excluded by ZL, and conflicts were resolved through discussion and consensus of the reviewers.

### Data charting

Data charting, akin to data extraction in systematic reviews, was independently performed by two reviewers (ZL & NA) to capture study information, including author, year of publication, study location, study type/ design, study participant demographics, aims of the study, outcome measures, and key results around barriers and facilitators influencing oral health access.

### Collating and summarising data

Two reviewers (ZL & NA) independently collated data. A structured three-step approach was utilised. First, an analytic framework was developed to map the key characteristics of the studies most relevant to the review, including population, study design, geographic distribution, and key findings. Next, data were thematically organised into barriers and facilitators affecting oral healthcare access. Here, the aim was to map key concepts rather than synthesising evidence as is common in systematic literature reviews. Lastly, a narrative account was developed to interpret findings and draw connections between identified themes.

### Theoretical framework

**Fig. 1:**
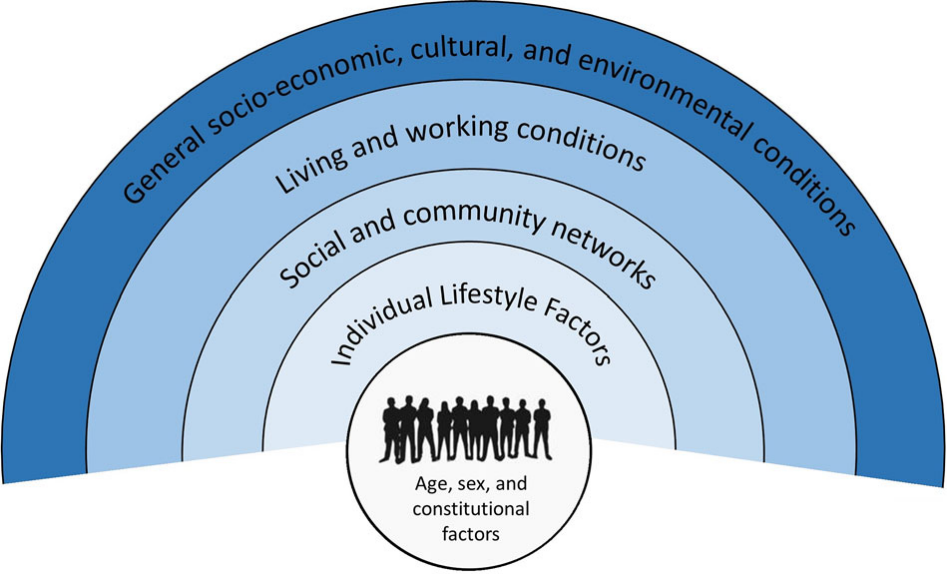
The Dahlgren and Whitehead model of health determinants [26].

This study employs the Social Determinants of Health model by Dahlgren and Whitehead (Fig. 1) to contextualise how multiple layers of social context shape access to oral healthcare [20, 25, 26]. The SDH model conceptualizes health as being influenced by a series of interrelated layers ranging from individual lifestyle factors to social and community networks, and extending to broader socio-economic, cultural and environmental conditions. We mapped barriers and facilitators identified in the included studies across these domains to illustrate how determinants at different levels interact to influence both oral health outcomes and access to oral healthcare services.

### Risk of bias and quality assessment

This scoping review addresses a broad and exploratory research question aimed at identifying barriers, facilitators, and socio-cultural influences on oral healthcare access. As the primary objective of a scoping review is to map and synthesise available evidence rather than assess its quality, conducting critical appraisal or risk of bias assessment is usually not recommended [22, 23, 27].

## Results

**Fig. 2:**
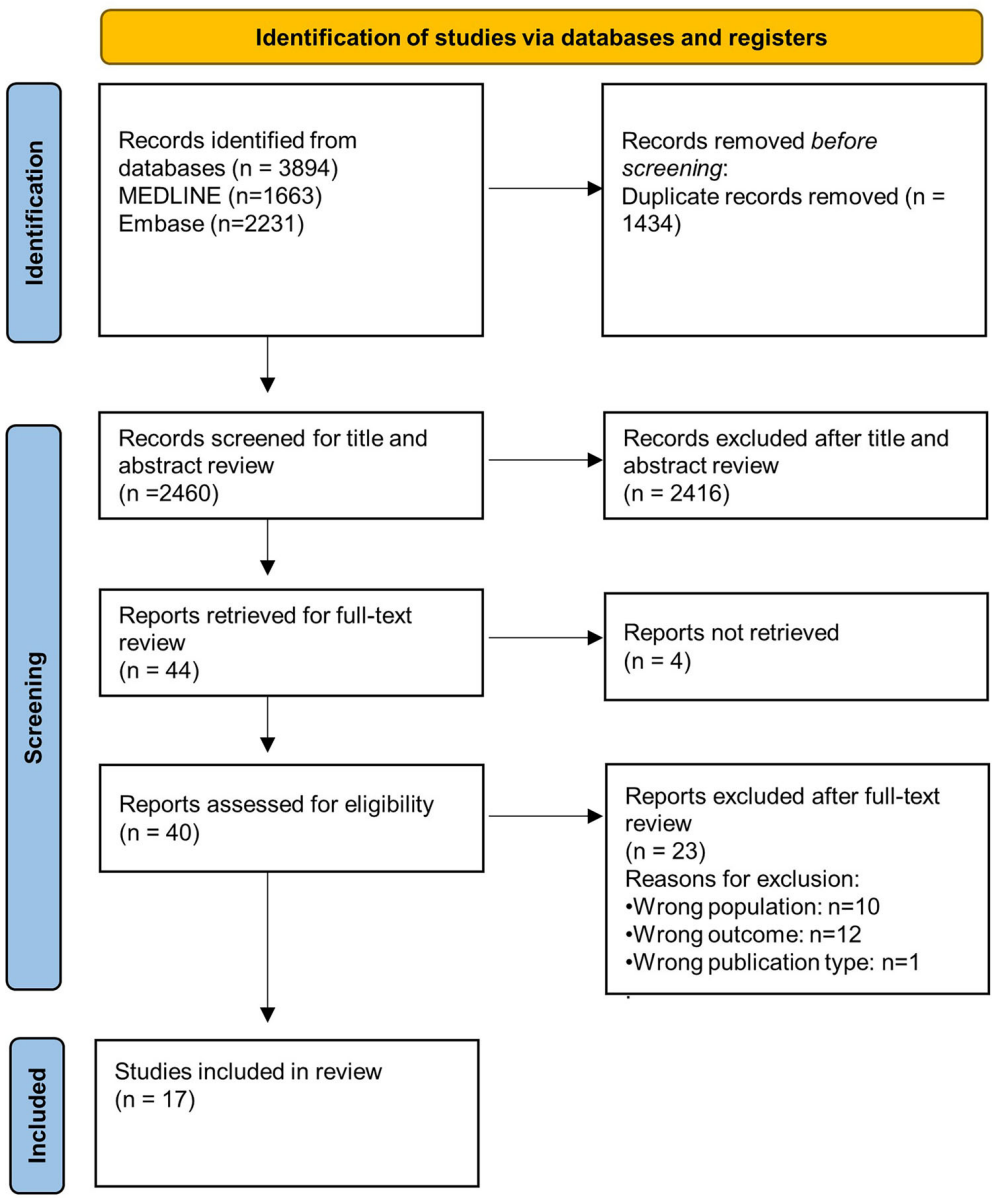
PRISMA Flow chart of search and screening process.

A total of 3894 articles were identified through database searches. After removing 1434 duplicates, 2460 records were screened for title and abstract. Of these, 44 articles were selected for full-text review; however, four could not be retrieved. Following full-text screening of the remaining 40 articles, 17 studies met the inclusion criteria and were included in the final review (see PRISMA flow diagram, Fig. 2).

Among the 17 included studies, 10 employed qualitative methods, 5 were quantitative cross-sectional surveys, and 2 utilised mixed-methods approaches. A total of 2653 participants across 8 HICs (USA, UK, Australia, Austria, Germany, Finland, Saudi Arabia and Canada) were included in the review.

**Fig. 3:**
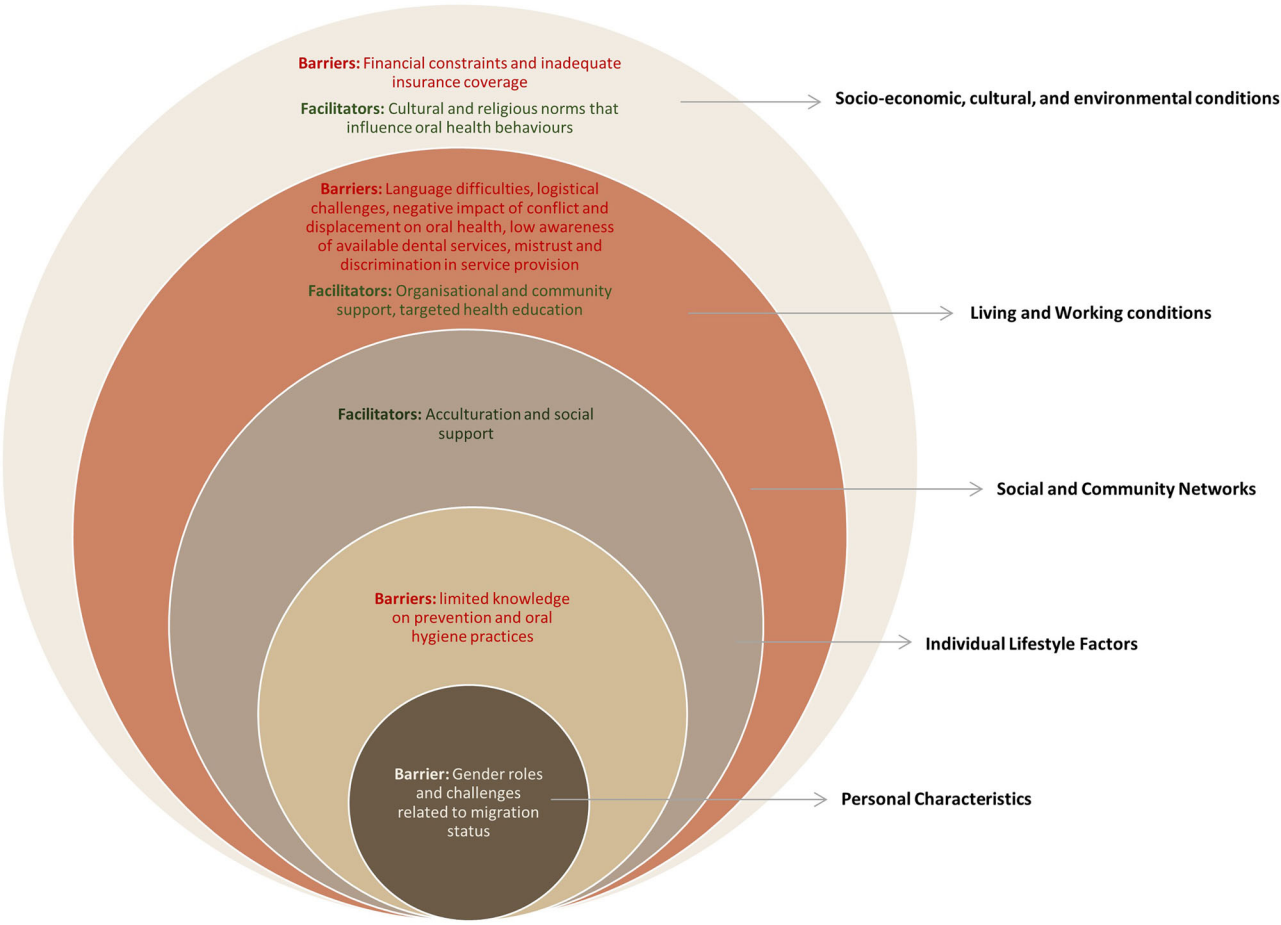
Social Determinants of Health Model highlighting barriers and facilitators to oral healthcare access.

An overview of the barriers and facilitators to oral healthcare access identified across the 17 studies are summarised in Fig. 3, using the Social Determinants of Health (SDH) model [26]. A more detailed account of these factors, along with the study characteristics, is provided in Supplementary Material 2.

1. *General, Socio-economic, Cultural, and Environmental Conditions*

This section represents the wider social, cultural, economic and environmental factors that impact access to oral healthcare and further health and well-being.

### Barriers

#### Financial constraints

12 out of 17 studies reported financial barriers as a major impediment to accessing oral healthcare among migrants experiencing vulnerabilities in HICs [9, 28–38]. Across studies, participants consistently described dental care as expensive, often prioritizing other basic needs over oral health despite recognising its importance [28, 33]. In several contexts, migrants perceived dental care to be less affordable and accessible in host countries compared to their home countries, however some studies noted that dental services were also costly in some countries of origin—Aldukhail et al., for example, noted that in Syria, dental services were only also costly and available only to those who could afford these [29, 32]. In a survey conducted in Germany, 82.6% (*n* = 540) of 724 participants agreed that financial barriers affected their ability to seek dental care [35]. Due et al. reported that Middle Eastern refugees in Australia delayed seeking dental help, citing both cost and normalisation of discomfort as contributing factors [31].

Dental care was also negatively affected by participants’ inability to cover household expenses, transportation costs, and the cost of oral hygiene products [9, 33]. Even where financial support was available—such as HC2 certificates in the UK, which provide help with health costs for individuals on low incomes, hidden expenses, including purchase of over-the-counter medications continued to pose challenges [34]. Additionally, Paisi et al. observed that asylum seekers and refugees often relied on cheaper, sugar-rich (cariogenic foods) due to financial constraints and limited affordability of healthier options, which may further contribute to tooth decay [9].

Four qualitative studies [31, 32, 39, 40] also highlighted the ways in which financial barriers led individuals to self-manage dental symptoms. Strategies reported in the studies included the use of over-the-counter painkillers, herbal remedies, and delaying care until symptoms became severe or unbearable.

##### Inadequate insurance coverage

Five studies addressing insurance coverage consistently reported ambiguity around the breadth of dental services included in insurance plans across different countries, leading to disrupted access to dental care [3, 29, 33, 36, 38]. In a US-based cross-sectional survey of 422 participants, 17% of Medicaid-insured participants were unaware of their dental coverage, which was significantly associated with unmet dental care needs (*p* = 0.02), and absence of dental cleaning in the past year (*p* < 0.001) [33]. Qualitative studies by Aldukhail et al. [29] in the United States and Keboa et al. [32] in Canada further highlighted limitations in insurance coverage, with participants reporting that inadequate coverage led to tooth extractions rather than comprehensive examinations or conservative management such as root canal treatment [29, 32].

### Facilitator

#### Cultural and religious norms

Four studies [9, 31, 38, 39] from Australia, the USA and the UK explored how cultural and religious norms shaped oral health practices and care-seeking behaviour. Three of these [6, 22, 23] highlighted oral health traditions in Islam, including the use of miswak, a chewing stick commonly used for oral hygiene. These studies described miswak as a culturally familiar practice with perceived therapeutic benefits among participants. Velez et al. [38] also reported that spirituality contributed to a positive health outlook, with several participants expressing that faith in a higher power provided emotional support when addressing oral health needs [38].

2. *Living and Working Conditions*

This layer the immediate environment and systems such as education, employment, training, transport, welfare services, housing, and other amenities that people interact with daily, which shape their access to oral health services.

### Barriers

#### Language difficulties

Language barriers were reported in seven studies [9, 29, 35–38, 40] as a challenge to both communication with dental providers and also participants’ ability to access care. In Germany, a cross-sectional survey found that 82.2% (*n* = 536) of refugees reported language difficulties as a major barrier [35]. Two studies [37, 38] noted challenges arising from reliance on interpreters, including misinterpretations or the interpreter answering in place of the professional, and concerns about information being lost in translation. Another study highlighted that women’s ability to schedule dental appointments often depended on their English proficiency, or having support from family, healthcare professionals, or community-based nurses who could assist in navigating the system and help make appointments on their behalf [36].

#### Logistical challenges

Five studies [28, 31, 32, 35, 41] identified logistical barriers that impeded access to dental care, including challenges with transport, and competing responsibilities such as work and childcare. Ponomarenko and Kaife [35] reported that participants with families were less likely to seek dental care (*n* = 335; 62.0%) compared to those who migrated alone (n = 55; 48.3%) [35]. Adeniyi et al. (2019) noted that affordable dental clinics were often located in less secure neighbourhoods, further deterring dental attendance [28]. Lastly, Keboa et al. [32] highlighted that humanitarian migrants in Montreal faced difficulties navigating the public transport system and adapting to harsh winter conditions, often leading to missed or delayed appointments [32].

#### Negative influence of conflict and displacement

Lamb et al. (2009) and Paisi et al. [9] both reported that the experience of conflict and displacement significantly influenced asylum seekers’ and refugees’ engagement with oral health care, which often fell to the bottom of their list of priorities [9, 39]. Immediate survival needs, including food, shelter, safety, and legal support—consistently took precedence over dental concerns, as reported in both studies conducted in Australia and the UK. Participants, as reported by a qualitative study in Australia, also acknowledged that deprioritising dental care due to chronic stress and trauma also contributed to dry mouth, further exacerbating the risk of tooth decay [39].

#### Low awareness of available dental services

Six studies [9, 30, 31, 34, 36, 37] highlighted limited knowledge about the availability and accessibility of dental care, which influenced their utilisation of services. In an Australian study, asylum seekers and refugees reported difficulty accessing relevant health information and confusion about eligibility for government dental schemes [31]. In the UK, participants in a qualitative study described registering with a dentist as different than with GPs and reported challenges finding providers willing to treat asylum seekers, resulting in care being sought only in cases of emergencies [34]. Another study also noted that language barriers exacerbated the lack of knowledge about where and how to access dental services [9].

#### Mistrust and discrimination in service provision

Mistrust in healthcare providers was a common barrier reported in eight studies [9, 28, 34, 35, 37–40]. Adeniyi (2019) and Velez et al. [38] both highlighted how asylum seekers, refugees and socially disadvantaged women reported feeling disrespected, stigmatised, or discriminated against during dental visits [28, 38]. In another study conducted in Austria, 7% of male participants explicitly cited mistrust as a reason for not accessing services [40]. Negative past experiences, for example, having a wrong tooth extracted, reinforced mistrust and led to reluctance to seek dental care [9, 38, 39]. Additionally, participants expressed frustration with perceived differences in healthcare systems, expecting more direct access to specialist care or proactive treatment and felt dismissed by providers [34]. Lastly, Riggs et al. [37] reported participants’ preference for private dental care sometimes because of mistrust in trainee dentists [37].

### Facilitators

#### Organisational and community support

Five studies [29–32, 37] described how support from various organisations facilitated access to dental care for migrants experiencing vulnerabilities. Sources of support included children’s schools, community dental clinics, migrant centres, and community members acting as interpreters and offering logistical support. Two studies [30, 37] highlighted the role of schools in prioritising children’s oral health and parental satisfaction with school dental health services amidst their economic constraints. This enabled children to receive regular dental-checkups, toothpaste and toothbrushes, and educational resources. Lastly, one study reported on the crucial role of government insurance offered through community dental clinics in Canada, which made dental care accessible for refugees who otherwise could not afford it [32].

#### Targeted health education

Three studies [18, 21, 26] reported that targeted health education initiatives improved oral hygiene behaviours and encouraged care-seeking among migrants. For instance, community-led educational programs in Montreal provided culturally sensitive oral health information that led to improved confidence in navigating dental systems [21]. Similarly, Adeniyi et al. [28] found that when dental education was integrated into maternal health services, it empowered women to prioritise oral hygiene and seek preventive care [26].

3. *Social and Community Networks*

Social and community networks encompass the wider social circles, support systems, and community connections that may positively or negatively affect access to oral healthcare. Within this section, we describe how acculturation and community support shapes oral health outcomes.

### Facilitators

#### Acculturation and social support

Two studies [31, 42] highlighted how adapting to a new healthcare system influenced oral health beliefs and help-seeking behaviours. Geltman et al. found that individuals with higher levels of acculturation were more likely to engage in preventive dental care, compared to those with low acculturation [42]. In Australia, Due et al. reported that social networks played an important role in encouraging dental help-seeking, with community members often acting as interpreters and providing logistical support to attend appointments [31].

4. *Individual Lifestyle Factors*

Individual lifestyle factors refer to personal behaviours such as oral hygiene practices, which can influence oral health status and the resulting need to seek care.

### Barriers

#### Limited knowledge on prevention and oral hygiene practices

Six studies [9, 30, 31, 34, 37, 39] identified that many migrants lacked adequate knowledge regarding preventive oral health measures, including unawareness of the importance of routine dental check-ups and confusion about proper oral hygiene techniques. For example, Riggs et al. [36] noted that refugee-background women often did not receive dental health information during antenatal care, and many were unaware of the link between pregnancy and increased oral health risk [36]. Similarly, Aldukhail et al. [29] reported that participants were unfamiliar with fluoride use and professional cleaning services, relying instead on traditional home remedies or irregular self-care practices [29].

5. *Personal Characteristics*

Personal characteristics, such as gender, migration status and other demographic characteristics, form the core of the SDH model and have a significant influence on health outcomes and access to care.

### Barriers

#### Gender roles

Five studies [9, 28, 30, 37, 40] reported that gender roles significantly influenced oral healthcare access among vulnerable migrant populations. Women, particularly mothers and pregnant individuals, were found to prioritise the health needs of their families over their own. For example, Riggs et al. [37] described how migrant mothers in Australia often delayed or avoided dental visits due to lack of childcare or the cultural expectation to focus on family wellbeing [37]. Adeniyi et al. [28] similarly found that socially disadvantaged pregnant women in Canada viewed oral health as a lower priority unless experiencing acute symptoms [28]. Paisi et al. [9] added that gender norms also influenced men’s dental behaviours, noting that some cultures discouraged men from expressing pain or seeking care. As one stakeholder explained, “Men often don’t express pain; they say ‘I’m fine’ even when they are not” [9].

#### Challenges related to migration status

Four studies [32, 35, 38, 40] identified legal status as a barrier affecting asylum seekers’ and refugees’ ability and willingness to access oral healthcare. Insecure migration status often limited entitlement to public health services or generated fear of interacting with formal systems. For instance, Ponomarenko and Kaifie [35] found that undocumented migrants in Germany were hesitant to seek dental care due to concerns about being reported to authorities [35]. Similarly, Keboa et al. [32] described confusion among humanitarian migrants in Canada about their eligibility for dental services, leading to missed opportunities for preventive care and a reliance on emergency treatments [32].

6. *Other barriers and facilitators:*

This section includes a factor that does not fit neatly into the predefined SDH layers but is a critical psychological barrier to care.

#### Fear and anxiety about dental treatment

Dental fear was identified as a psychological barrier in five studies [28, 35, 38, 39, 43]. Participants expressed anxiety about potential pain during procedures, distrust in unfamiliar healthcare settings, and traumatic prior experiences. Velez et al. [38] found that fear of dental pain was a significant deterrent for Mexican migrant women, especially when combined with previous negative encounters in both their home countries and host nations [38]. This was noted by Lamb et al. (2009), where Afghan refugees described oral healthcare as invasive and were reluctant to attend unless absolutely necessary [39].

## Discussion

We conducted a scoping review to map barriers, facilitators, and socio-cultural factors influencing access to oral healthcare access among vulnerable migrant groups in HICs and found a range of individual, structural, and systemic barriers. Previous reviews conducted by Paisi et al., Keboa et al., Lauritano et al., and Zinah et al. [2, 3, 16, 18] have established that asylum seekers and refugees (ASRs) experience a higher burden of oral diseases and significant challenges to accessing services compared to the host population. Our review adds to this evidence by synthesizing findings from 17 studies across a broader range of vulnerable migrant groups beyond ASRs and integrating both qualitative and quantitative data. Specifically, we map factors such as: financial constraints, language difficulties, mistrust and discrimination in service provision, logistical challenges, cultural and religious norms, and limited knowledge on prevention, demonstrating how these operate across various individual, community, and broader societal domains within the SDH framework.

Consistent with previous literature, high costs of dental treatment, limited insurance coverage and a lack of clarity around entitlements, especially among ASRs, were identified as significant barriers to care [9, 29, 32, 33, 37, 38]. This finding situates oral health in the broader economic sphere, suggesting that dental care is often perceived as a luxury rather than a necessity, and is overlooked in favour of other basic needs. This further leads to poorer oral health outcomes and a higher burden of disease among migrants compared to the host population, driven by limited accessibility, high-sugar diets, and inadequate resources to maintain oral hygiene [3, 15, 18].

Language barriers and unfamiliarity with new healthcare systems also worsened access difficulties by hindering communication with providers and reducing health-seeking behaviours [29, 35–37, 40]. Preventive dental care was generally uncommon, and participants often sought care only when experiencing severe symptoms—reflecting both limited awareness of preventive strategies and cultural differences in perceptions of routine dental check-ups [36, 37]. This practice of only seeking emergency care is a reflection of not just individual preferences, but also a wider systemic failure where access is constrained by lack of availability of interpreters and administrative complexity in navigating healthcare. We found that acculturation significantly influenced these behaviours and individuals with higher levels of acculturation were more likely to engage in preventive dental care compared to those less adapted to the new cultural environment [31, 42]. This shift suggests that successful integration can positively impact health-seeking behaviours and awareness of preventive practices, a pattern also highlighted by Dahlan et al. [44].

Mistrust toward dental professionals and the broader healthcare environment was another recurring and profound theme. Studies reported on medical negligence—such as wrong-tooth extractions and previous negative interactions contributing to long-term avoidance of dental services [9, 28, 35, 39]. Mistrust in healthcare was exacerbated by stigma, perceived lack of respect, and differential treatment based on race, language, class status and migration background [38]. These findings point to systemic failures within the service delivery system consistent with broader literature on institutional racism and structural violence, where negative experiences lead to avoidance of services, non-compliance and worsening of health outcomes [45]. Fear and anxiety associated with seeking care are a direct, psychological consequence of these systemic failures [9, 18, 46].

Logistical barriers further discouraged dental visits. Studies suggested that as a consequence of financial constraints, individuals were forced to seek dental care in remote and unsafe locations to save costs. Transportation challenges and the need for childcare, especially among single mothers, also emerged as a prominent barrier. These issues may help explain findings that migrants with families were less likely to visit a dentist than those migrating alone [35], suggesting that family responsibilities and scarcity of resources interact to create compounding logistical barriers.

Notably, only a few prior reviews have explored how demographic variables such as immigration status, gender, and cultural or religious norms influence oral care access [2, 3, 18]. This review highlights that women often report the highest levels of unmet healthcare needs, which may be influenced by either individual characteristics and choices as well as broader socio-cultural gender norms [40]. Many women were more likely to prioritise the health of family members over their own and postpone dental care until experiencing acute symptoms. Literature further suggests that women generally hold more positive attitudes towards dental visits, possess greater oral health literacy, and engage in better oral hygiene practices than men [47]. Counterintuitively, their higher level of unmet needs may therefore reflect societal circumstances that prevent them from fully exercising their knowledge and literacy in practice. In contrast, cultural expectations often discourage men from expressing pain or seeking care, a finding supported by Lipsky et al. [48].

Culture, religion and spirituality also played dual roles. The use of miswak was framed as a facilitator in several studies due to its perceived therapeutic value; however, the broader evidence is mixed regarding its clinical efficacy and potential drawbacks, such as abrasiveness [49, 50]. Importantly, cultural and religious practices do not necessarily facilitate access to dental care directly; rather, they influence oral health behaviours and may shape perceived need for care, which then interacts with structural and systemic barriers identified in this review. In some studies, religion and spiritual beliefs promoted a positive outlook towards oral health, consistent with findings that religiosity can have a protective effect on dental caries [51]. However, another study conducted in Germany reported that religious affiliations can lead to a lower oral health-related quality of life, potentially due to reliance on religious beliefs for medical decision making [52]. These results emphasise the importance of cross-cultural knowledge, cultural humility and recognition of religious perspectives in developing culturally sensitive care and improving provider-patient relationships.

### Strengths and limitations

This scoping review represents the first comprehensive synthesis examining the barriers, facilitators, and socio-cultural factors influencing oral healthcare access among all categories of migrants that experience vulnerabilities - not limited to ASRs—in HICs. This study employed the multilevel SDH model to structure its analysis, enabling a nuanced understanding of access issues across individual, community, and broader societal levels. The review included both qualitative and quantitative data, which reinforced the consistency of findings across diverse geographic and policy contexts. These findings align with broader literature on healthcare integration and access, such as the work by Harnagea et al. [53], which emphasised the importance of theoretical models in mapping barriers and facilitators to oral health integration in primary care [53].

While this scoping review offers valuable insights, it is important to acknowledge its limitations. Firstly, while the terminology ‘vulnerable migrants’ was adopted for consistency with operational definitions used by OHID (UK) [1], we recognise it is inherently reductive and not person-first. We also acknowledge that findings may not be transferable to medium and low-income countries, where most vulnerable migrants are hosted. Additionally, all migrants, whether migrating with full voluntary agency or under constrained circumstances, may experience some degree of disadvantage and vulnerability during or after their journey. This may stem from challenges such as cultural adaptation, navigating new systems, or how welcoming a new country is. However, the nature and degree of vulnerabilities may vary considerably between migrants. Future research should therefore avoid lumping migrants as a homogenous group, as this risks missing disparities affecting those with least agency and the highest levels of vulnerability.

Secondly, no formal quality appraisal or risk of bias assessment of the included studies was conducted, which may affect reliability of synthesised findings, although it is consistent with scoping review methodology, which prioritises breadth of evidence to map existing body of literature [22, 23, 54].

Finally, the heterogeneity of healthcare systems across HICs - such as the publicly funded National Health Service (NHS) in the United Kingdom versus the predominantly insurance-based system in the United States - presents challenges in drawing direct comparisons across contexts. These systemic differences underscore the complexity migrants face in navigating unfamiliar healthcare landscapes, often compounded by legal, linguistic, and cultural barriers [9]. Future research may benefit from focusing on more homogenous healthcare environments to allow for generalisability and transferability of findings across similar systems and to better isolate and understand the specific dynamics influencing access. Alternatively, studies could employ comparative narrative syntheses to capture how structural and contextual factors differ across various settings.

### Implications for policy and practice

This review has direct implications for dental policy and service design aimed at promoting equitable oral healthcare access for vulnerable migrants in high-income countries (HICs). By applying the Dahlgren and Whitehead model of the Social Determinants of Health, the findings can be conceptualised across multiple levels of influence.

At the broader societal level, policymakers must address structural barriers by broadening insurance coverage to mandate comprehensive dental care, simplify legal documentation processes and integrate oral health into national public health strategies. Oral health should be recognised not merely as a clinical issue but as a public health priority with significant implications for overall well-being and social exclusion [9].

At the community level, collaboration between dental professionals, local authorities, and community organisations is essential. Efforts should focus on clarifying appointment systems, improving emergency dental care access, and disseminating culturally sensitive oral health information. Community-based educational interventions have shown promise in bridging gaps in access and improving health literacy [3].

At the individual level, migrants should be supported in understanding their healthcare entitlements, particularly in complex or fragmented systems such as those in the U.S. and parts of Europe. Community resources and advocacy groups can play a pivotal role in this regard. Furthermore, increased participation of migrants in health research is vital to capture lived experiences and evaluate the effectiveness of interventions [55, 56].

## Conclusion

This review mapped a complex network of individual and systemic barriers—ranging from, but not limited to, financial and logistical constraints, mistrust and cultural dissonance—that limit oral healthcare access for vulnerable migrants. By examining these factors through the lens of the Social Determinants of Health model, our findings underscore that access barriers are rooted not merely in individual behaviours, but in structural and intermediary determinants of health. The persistence of these barriers necessitates a shift toward a holistic, equity-oriented approach that prioritises structural and systemic reform over individual-level interventions. Policies and practices must evolve toward inclusive, culturally competent, and accessible dental care systems to ensure that all population groups, especially the most vulnerable, benefit from optimal oral health.

## Supplementary information

Supplementary Data

## Data Availability

All data analysed during this study are included in this manuscript and its supplementary information.
