# Supplementary material for: Mapping the barriers and facilitators of oral healthcare access for vulnerable migrants across high-income countries: a scoping review
Source: BDJ Open. 2026 Feb 13;12:17. doi: 10.1038/s41405-026-00398-0 (PMC12904860; doi:10.1038/s41405-026-00398-0)
Supplement: Supplementary file 1 — Supplementary Data [file 41405_2026_398_MOESM1_ESM.docx]

**Mapping the Barriers and Facilitators of Oral Healthcare Access for vulnerable Migrants across High-Income Countries: A Scoping Review**

**Supplementary Data**

**Supplementary Material 1. Search Strategies for Ovid MEDLINE and Embase**

**Ovid MEDLINE(R) ALL <1946 to April 30, 2024>**

1 "transients and migrants"/ 14763

2 migrant*.mp. 31069

3 expatriate.mp. 784

4 departee*.mp. 1

5 asylum.mp. 4877

6 foreign-born.mp. 4282

7 foreign born.mp. 4282

8 foreign worker.mp. 68

9 foreign student.mp. 33

10 international student.mp. 541

11 sex traffick*.mp. 453

12 ((wom?n or child* or man or men) adj2 traffick*).mp. 328

13 Refugees/ 13780

14 (forced adj3 migrat*).tw,kf. 634

15 (displaced adj3 (person* or people or population*)).tw,kf. 2079

16 immigrant*.mp. 36863

17 exp Human Migration/ 28094

18 or/1-17 95801

19 "head and neck neoplasms"/ or "squamous cell carcinoma of head and neck"/ or facial neoplasms/ or exp mouth neoplasms/ 147889

20 ((oral or mouth or head or neck or face or facial or oropharyngeal or tongue or saliva* or gum or lip or gingival or palate) adj5 (tumo* or cancer* or neoplas* or carcino* or malign* or benign*)).mp. 216243

21 Oral Health/ 21201

22 Dental Caries/ 50930

23 exp Dental Care/ 35343

24 exp Tooth Diseases/ 189442

25 exp Dentists/ 21359

26 (oral adj3 (health or hygiene or care)).tw,kf. 54652

27 dental.tw,kf. 282432

28 (tooth adj3 (health or hygiene or care)).tw,kf. 742

29 (teeth adj3 (health or hygiene or care)).tw,kf. 1086

30 dentist*.tw,kf. 94259

31 oral hygiene/ 14015

32 (dental adj3 (health or hygiene or care)).tw,kf. 32924

33 (mouth adj3 (health or hygiene or care)).tw,kf. 865

34 Dental Health Services/ 4201

35 dental health service*.tw,kf. 803

36 oral health service*.tw,kf. 979

37 Dental Hygienists/ 5977

38 dental hygienist*.tw,kf. 3164

39 exp Periodontal Diseases/ 97695

40 periodontal disease*.tw,kf. 28693

41 exp Periodontitis/ 35996

42 periodontitis.tw,kf. 37082

43 Gingival Diseases/ 4778

44 gingival disease*.tw,kf. 347

45 exp Gingivitis/ 12217

46 gingivitis.tw,kf. 9561

47 (oral adj2 infect*).tw,kf. 7264

48 exp Leukoplakia, Oral/ 4055

49 leukoplaki*.tw,kf. 5601

50 Oral Submucous Fibrosis/ 1113

51 oral submucous fibrosis.tw,kf. 1726

52 oral ulcer/ 2582

53 ((oral or mouth) adj2 ulcer*).tw,kf. 4745

54 or/19-53 772405

55 18 and 54 1663

**Embase <1974 to 2024 April 30>**

1 exp migration/ 51778

2 migrant*.mp. 35904

3 exp migrant/ 51229

4 expatriate.mp. 868

5 departee*.mp. 1

6 asylum.mp. 5756

7 (foreign-born or foreign born).mp. 5168

8 foreign worker.mp. 4599

9 foreign worker/ 4561

10 foreign student.mp. or foreign student/ 329

11 international student.mp. 487

12 sex traffick*.mp. 651

13 ((wom?n or child* or man or men) adj2 traffick*).mp. 393

14 exp refugee/ 18430

15 (forced adj3 migrat*).tw,kf. 633

16 (displaced adj3 (person* or people or population*)).tw,kf. 2150

17 immigrant*.mp. 40984

18 or/1-17 125536

19 "head and neck tumor"/ 18303

20 "head and neck squamous cell carcinoma"/ 23634

21 face tumor/ 3250

22 mouth tumor/ 14912

23 ((oral or mouth or head or neck or face or facial or oropharyngeal or tongue or saliva* or gum or lip or gingival or palate) adj5 (tumo* or cancer* or neoplas* or carcino* or malign* or benign*)).mp. 283851

24 dental caries/ 57274

25 dental procedure/ 34858

26 exp tooth disease/ 249010

27 exp dentist/ 30166

28 (oral adj3 (health or hygiene or care)).tw,kf. 58643

29 dental.tw,kf. 272112

30 (tooth adj3 (health or hygiene or care)).tw,kf. 838

31 (teeth adj3 (health or hygiene or care)).tw,kf. 1050

32 dentist*.tw,kf. 88677

33 mouth hygiene/ 33374

34 (dental adj3 (health or hygiene or care)).tw,kf. 32893

35 (mouth adj3 (health or hygiene or care)).tw,kf. 1067

36 dental health service*.tw,kf. 844

37 oral health service*.tw,kf. 994

38 dental hygienist/ 907

39 dental hygienist*.tw,kf. 3020

40 exp periodontal disease/ 120594

41 periodontal disease*.tw,kf. 29707

42 exp periodontitis/ 54758

43 periodontitis.tw,kf. 39227

44 exp gingiva disease/ 42589

45 gingival disease*.tw,kf. 373

46 exp gingivitis/ 19816

47 gingivitis.tw,kf. 9879

48 (oral adj2 infect*).tw,kf. 8656

49 exp oral leukoplakia/ 768

50 leukoplaki*.tw,kf. 5887

51 oral submucous fibrosis/ 364

52 oral submucous fibrosis.tw,kf. 1674

53 mouth ulcer/ 15714

54 ((oral or mouth) adj2 ulcer*).tw,kf. 8072

55 or/19-54 808315

56 18 and 55 2231

**Supplementary Material 2. Overview of included studies**

| **Study** | **Country** | **Study Design** | **Study aim(s)** | **Participants** | | | | **Key Results** |
| --- | --- | --- | --- | --- | --- | --- | --- | --- |
| **(Author, Year)** |  |  |  | **Sample (N, Population)** | **Age** | **Gender** | **Race/ Ethnicity/ Nationality/ Country of Origin** |  |
| **Adeniyi (2019)** | Canada | Qualitative | To explore the perceptions of a sample of socially disadvantaged women on oral healthcare provision during pregnancy | 17, socially disadvantaged women | 31.4 years | All female (100%) | White, South Asian, Asian, Aboriginal, Metis, Indian, Mixed (Filipino - Canadian), and African | **Financial constraints:** lack of dental insurance and high costs led to dental care being deprioritised  **Logistical challenges:** remote and unsafe clinic locations discouraged service utilisation  **Mistrust of healthcare providers:** participants felt stigmatised by providers due to incarceration and substance use history |
| **Aldukhail (2023)** | USA | Mixed methods | 1) To qualitatively describe the oral and emotional health challenges experienced by the sample of refugees across different stages of resettlement.   2) To quantitatively describe the status of oral and emotional health for refugees in Massachusetts  3) To assess the access to dental care for refugees after arriving in the United States for future needs assessment. | 81 (Quantitative survey participants **n=69**; qualitative interview participants **n=12**), refugees | **Quantitative study:** 25-44 years  **Qualitative study:** 21-66 years | **Quantitative study:** 24 males and 23 females  **Qualitative study:** 7 males and 5 women  females | Syrian (60%), Iraq (31.1%) and Afghanistan (8.9%) | **Financial constraints:** cost of private dental care and long wait lists discouraged participants from accessing services in their home countries.  **Insurance coverage:** gaps in insurance coverage led to tooth extractions as conservative treatment options were not covered.  **Language:** reported as the most common barrier (32.6%) to scheduling appointments. |
| **Carrion (2011)** | USA | Qualitative | To understand factors that impact dental service utilization | 48, migrant farmworkers | Not specified | Female - 77% and Male - 23% | Not specified | **Financial constraints:** lack of financial resources, insurance and unavailability of services deterred care until mobile dental units were available.  **Lack of awareness of services:** parents lacked knowledge of emergency dental options for children.  **Support from community organisations:** The Migrant Head Start Center provided children and families with toothbrushes, toothpaste, and oral health education, which helped parents treat their children’s dental health as a priority despite their economic constraints and limited resources. |
| **Due (2020)** | Australia | Qualitative | To improve understanding of Middle Eastern refugees' and asylum seekers' oral health help-seeking and to determine the utility of Andersen's Model in this context. | 20, refugees (n=17) and asylum seekers (n=3) | 18-52 years (mean – 32.85) | 8 Males and 12 Females | Syria (n=11), Iran (n=3), Afghanistan (n-6) | **Financial constraints:** cost of services and long wait times were reported as barriers to accessing care.  **Logistical:** single mothers reported transport barriers while accessing care, overcome by social networks.  **Self-management:** in the absence of social support, some resorted to home remedies instead of seeking professional care.  **Acculturation:** Familiarisation with Australian health norms and systems of care led to a change in oral health beliefs and help-seeking behaviours.  **Social networks:** Community members helped as interpreters and provided logistical support, facilitating dental help-seeking for refugees and asylum seekers. |
| **Geltman (2013)** | USA | Cross-sectional survey | To investigate the impact of English health literacy and spoken proficiency and acculturation on preventive dental care use among Somali refugees | 439, refugees | 35.2 years | 58.1% - female | Somali (n=382, 87%), Somali Bantu (n=57, 13%) | **Facilitator: acculturation:**  Individuals with high acculturation levels were 2.8 – 3.8 times more likely to use preventive dental care than those with low acculturation.  **Education and health literacy:** participants with higher functional health literacy were 2 times more like to seek preventive dental care than those with lower levels of literacy. |
| **Keboa (2019)** | Canada | Qualitative - focused ethnography | To understand the oral healthcare experiences of humanitarian migrants in Montreal and their perceptions of ways to improve access to oral healthcare | 25, humanitarian migrants (13 refugees and 12 asylum seekers) | 18-35 years: 13  ≥ 36 years: 12 | 9 males, 16 females | Latin America (n=5), North Africa and Middle East (n=5), Sub-Saharan Africa (n=12). | **Financial constraints:** high cost of treatment led to participants to opt for extractions over restorative care like root canal treatments.  **Logistical:** transport challenges, weather conditions and psychosocial stress contributed to missed appointments. |
| **Kohlenberger (2019)** | Austria | Cross-sectional survey | To explore: 1) refugees’ subjective well-being, 2) their access to health care providers and most frequent barriers for service utilization and 3) their satisfaction with the quality of health care provision. | 515, refugees | 18-61 years | 73 females, 447 males | 54% Syrian, 16% Iraqi, 23% Afghan, 7% other citizenship | **Language:** 11-12% of participants experienced language barriers and had insufficient knowledge of suitable healthcare providers.  **Self-management:** the most common reason for not seeking care was waiting for the condition to improve without treatment (21-22%). |
| **Lamb (2009)** | Australia | qualitative descriptive study | To gain an in-depth understanding about oral health from one group of highly vulnerable refugees | 8, refugees | 20-65 years | 3 males and 3 females | Hazara refugees, an ethnic minority from Afghanistan | **Self-management:** participants delayed seeking care until pain became severe, traditional remedies like herbs and cloves used as interim solutions.  **impact of conflict and displacement:** safety, instability and lack of access to hygiene resources affected daily care routines.  **Mistrust:** fear of poor outcomes and negative perceptions of providers led to avoidance of dental care. |
| **Leopold (2021)** | USA | Cross-sectional survey | To identify predictors of access to dental health care among drivers | 422, low paid migrant workers | 19-55+ years | Males: 411  Females: 11 | South Asia: 36% (Bangladesh, India, Pakistan, Nepal). Latin America: 20% (e.g., Brazil, Colombia, Ecuador). Sub-Saharan Africa: 19% (e.g., Nigeria, Ghana, Sudan). North Africa/Middle East/Central Asia: 10% (e.g., Afghanistan, Morocco, Turkey). East Asia/Tibet/Southeast Asia: 5% (e.g., China, South Korea, Tibet). Other regions: 10%. | **constraints:** Difficulty covering household expenses significantly associated with lower odds of accessing care (OR = 0.5; 95% CI: 0.28–0.89; p < 0.05).  17% of Medicaid-insured participants unaware of their dental coverage, significantly associated with:   - Inability to access needed care (p = 0.026). - Lack of past-year dental cleaning (p < 0.001). |
| **Mattila (2016)** | Finland | Cross-sectional survey | To investigate self-reported oral health, oral health habits, dental fear and use of dental health care services among asylum seekers and immigrants in Finland. | 38, asylum seekers and immigrants | 17-53 years | 18 males and 20 females | Participants originated from 15 countries across Asia, Africa, and Europe: Asia: 21 participants (all asylum seekers and 12 immigrants). Africa: 7 immigrants. Europe: 10 immigrants. | **Difficulty with appointments:** all asylum seekers (100%) and half of immigrants (50%) found it difficult to obtain appointments. AS were also significantly more dissatisfied with access to care (p=0.003) and quality of treatment (p=0.001) compared to immigrants. |
| **O'Donnell (2007)** | UK | Qualitative | To identify the barriers and facilitators to accessing health care, both medical and dental, and to explore the health care needs and beliefs of asylum seekers living in one part of the UK. | 52, asylum seekers | 20-57 years | Females: 31 Males: 21 | Africa: DRC, Zimbabwe, Somalia, Guinea. Middle East/South Asia: Afghanistan, Iran, Lebanon, Pakistan, Sri Lanka. Europe: Albania, Azerbaijan, Russia, Turkey. | **Limited awareness of services:** Registration process was unfamiliar compared to GP services and participants struggled to find providers willing to treat asylum seekers.  Participants expected specialist referrals or easier access to antibiotics and were disappointed when these were not provided. |
| **Paisi (2022)** | UK | Qualitative | To investigate factors influencing oral health behaviours and access to dental services for asylum seekers and refugees (ASRs) | 12, refugees and asylum seekers | Not specified | Males: 4 Females:8 | diverse ethnic backgrounds, but specific racial/ethnic demographics not specified | **Financial constraints:** ASRs struggled with cost of treatment, transport, and purchasing oral hygiene products. They also relied on cheaper, more sugary food, which worsened their dental problems.  **Difficulty with appointments:** early morning calls for appointments and limited phone access made accessing dental care difficult. Further, limited availability of interpreters and culturally appropriate care created additional obstacles.  **Impact of conflict and displacement on oral health:** oral health was deprioritised due to more pressing needs such as food, housing, and legal concerns.  **Mistrust in healthcare:** past negative or traumatic dental experiences affected ASRs willingness to seek dental care. |
| **Pani (2017)** | Saudi Arabia | Mixed methods | To use the P-CPQ-8 (Parental-Caregiver Perceptions Questionnaire-8) questionnaire to quantitatively assess OHRQoL (Oral health-related quality of life) among the parents of children aged below 6 years and then use a focus group interview to explore the factors influencing their perceptions of their children's oral health. | 42, refugee parents | 38.2 years (±8.1 years) | Not specified | Syrian (100%) | **Logistical challenges:** fear of taking time off work, inability to balance work and childcare, and cost of transport prevented access to dental care. |
| **Ponomarenko (2023)** | Germany | Cross-sectional survey | To evaluate oral health and hygiene of Ukrainian refugees, identify barriers accessing dental health care and explore the relation to their mental health state. | 724, refugees | Mean: 37.5 years | males - 78, females - 640 | Ukranian (100%) | **Financial and language barriers:** finance and language barriers were reported by 82.6% (n=540) and 82.2% (n=536) of participants.  **logstical:** participants with families were less likely to visit a dentist (n= 335, 62%) when they needed one compared to those who migrated to Germany alone (n=55, 48.3%)  **mistrust in healthcare:** 74.5% of participants with unsuccessful consultations rated their teeth in poor/ very poor condition, vs. 54.9% among others.  75.9% of the same group reported poor gum health, vs. 58.1% of others. |
| **Riggs (2014)** | Australia | Qualitative | 1)To explore the dental service utilization and experiences of migrant mothers from Iraq, Lebanon, and Pakistan in Melbourne, Australia.  2) To identify barriers and enablers to accessing mainstream dental services for migrants. | 122, migrants | Not specified | All female (100%) | Migrant mothers from: Iraq (including Assyrian Chaldean background) Lebanon Pakistan | **Language barrier:** Language barriers and reliance on interpreters created challenges in communication with dental providers as participants believed interpreters lacked dental knowledge leading to misunderstandings.  **Financial constraints:** high cost of private care discouraged participants from seeking care. There was confusion about which services were free and what required payment.  **Difficulty with appointments:** dental waiting lists were lengthy, causing frustration and anxiety, especially for those experiencing pain.  **Limited awareness of prevention:** care was sought during emergencies, with preventive visits, especially for children being rare. |
| **Riggs (2016)** | Australia | Qualitative | To describe Afghan and Sri Lankan women's knowledge and beliefs surrounding maternal oral health, barriers to accessing dental care during pregnancy, and to present the perspectives of maternity and dental service providers in relation to dental care for pregnant women | 27, refugees and migrants | Not specified | 24 Females, 3 Males | 14 Afghan, 11 Sri Lankan | **Language & dependency:** care-seeking often depended on English proficiency or access to an English-speaking family member or health worker to navigate the system.  **Insurance coverage:** Participants were unaware they qualified for cost exemptions under refugee/ asylum schemes, and unclear on what was covered.  **Difficulty with appointments:** Being placed on waiting lists without follow-up caused disengagement and reluctance to recontact services.  **Limited awareness of prevention:** Across both cultural groups (Afghan and Sri Lankan), preventive care was not routinely pursued, dental visits were primarily for symptomatic issues. |
| **Velez (2017)** | USA | Qualitative | To qualitatively examine facilitators and barriers to dental care access and quality services among Mexican migrant women | 52, migrants | 18-81 years | All female (100%) | Mexican (100%) | **Language barrier:** lack of  access to Spanish-speaking staff affected ability to  obtain dental care. Appointment avoidance was noted if providers only spoke English.  **Immigration status:** fear of legal exposure and inability to travel to return to Mexico for affordable care discouraged service use.  **Trust & respect:**  Participants felt that there was a lack of  respect from providers and supporting dental office staff towards patient, undermining trust.  **discrimination:** Perceived differential treatment based on race, language and class status, and type of insurance coverage  **medical negligence:** A recurring concern among Lideres was providers’ lack  of responsibility and medical negligence. |
